# Dipyrone and blood dyscrasia revisited: "non-evidence based medicine"

**DOI:** 10.1590/S1516-31802005000300001

**Published:** 2005-05-02

The LATIN study (Incidence of aplastic anemia and agranulocytosis in Latin America) is a Latin American multicenter study to estimate the incidence of aplastic anemia and agranulocytosis in Latin America. We now have the results from the Brazilian pilot phase including data from seven centers in the states of Paraná, Goiás, Minas Gerais, Amazonas, Pernambuco and São Paulo.^1^ During the pilot study, 16 cases of agranulocytosis and 74 cases of aplastic anemia were included with a total incidence of 0.5 cases per million individuals per year for agranulocytosis and 2.7 cases per million individuals per year for aplastic anemia.

In a previous Brazilian study in the Southern region, the incidence of aplastic anemia observed was 2.4 cases/million individuals per year, which was very similar to the present data. The incidence of aplastic anemia reported from studies in Mexico and Thailand was higher: 3.9 and 4.1 million individuals/year, respectively. However, even with higher values, the incidence of aplastic anemia in Mexico and Thailand can be considered to be low in comparison with other countries. The same pattern has also been described when agranulocytosis is considered as an isolated endpoint.^2^

One frequent cause of agranulocytosis is prescription and use of over-the-counter drugs. The main drugs associated with agranulocytosis have been antithyroid drugs, sulfasalazine, sulfamethoxazole-trimethoprim, clomipramine hydrochloride, dipyrone with analgesics, the penicillin group, cimetidine, phenylbutazone, penicillamine and indomethacin.^3,4^

Dipyrone is widely used worldwide as an analgesic, including in Europe and Latin America. In the United States, it has been banned because of a possible association with agranulocytosis, and it is known there by the derogatory nickname of "Mexican aspirin".^5,6^ In 2001, media pressure caused a debate in Brazil. In a symposium that included Brazilian and international researchers, it was concluded that dipyrone was being used as an over-the-counter medication.^7^ One year later, "The Lancet" published an editorial discussing a Swedish study with excellent methodology that demonstrated a positive association between dipyrone and agranulocytosis. The editorial also said that, although the study was based on a small number of cases, it would indicate an epidemic of agranulocytosis if these numbers were translated to countries in which dipyrone is commonly used.^8^

The editorial concluded that more studies needed to be done in countries where dipyrone is commonly used as an over-the-counter medication. This is the target that the "Latin Study" is addressing. It is very important to ascertain whether dipyrone is really associated with large numbers of agranulocytosis cases. Nevertheless, dipyrone is a cheap analgesic with great power to reduce pain. Consequently, it is a first-line medication for treating pain in countries like Brazil, Argentina, Mexico, Spain and others.

From a personal point of view, I have no conflict of interest in this matter, with regard to grants, fees, stocks, or employment relationships with pharmaceutical companies that manufacture dipyrone. However, I am a long-term migraine sufferer and, as a physician, I have also treated a lot of migraines during my professional career. I can testify that it is very good to have dipyrone available for prescribing to those who are suffering pain. Nonetheless, the truth needs to be known. Some papers indexed on Medline have described dipyrone as the Mexican aspirin and have discussed the possible consequences of dipyrone use in such a way as to provide a very good example of non-evidence based medicine. This is just prejudice, not science.

Brazil has discussed the idea of removing dipyrone from sale as an over-the-counter medication. Why not discuss other drugs such as acetaminophen? In many countries, acetaminophen is not an over-the-counter medication because of the risk of suicide among people using it. Acetaminophen can cause hepatic necrosis in high doses. Thus, countries like the United Kingdom changed their legislation on analgesic pack sizes in 1998, so as to reduce the packs sold over-the-counter.^9-12^ Other studies have shown an increased risk of suicide from over-the-counter sales: older women^13^ and young adults that try suicide for the first time are some of the risk groups.^14^

If we enter Medline and search for dipyrone and toxicity, we can find 85 references. If we do the same for acetaminophen, we can find 1,661 references; and if we do the same for aspirin, we can find 1,663 references (Medline was accessed on Febrary 22, 2005). What is the message? If we wish to say something about a medication, or anything else, we have to do so on the basis of evidence. The Latin Study may answer this old question about dipyrone and agranulocytosis. This is the correct way to solve this problem. Attitudes cannot be adopted on the basis of impressions, prejudice, economic interests, media pressure or other reasons. Attitudes need to be based on facts. It is very important for everyone to know about the results of the Latin Study!
